# Computerized tomography myelography with coronal and oblique coronal view for diagnosis of nerve root avulsion in brachial plexus injury

**DOI:** 10.1186/1749-7221-2-16

**Published:** 2007-07-25

**Authors:** Hiroshi Yamazaki, Kazuteru Doi, Yasunori Hattori, Sotetsu Sakamoto

**Affiliations:** 1 Advanced Emergency and Critical Care Center, Shinsyu University Hospital, Matsumoto, Nagano, Japan; 2 Department of Orthopedic Surgery, Ogori Daiichi General Hospital, Ogori, Yumaguchi, Japan

## Abstract

**Background:**

The authors describe a new computerized tomography (CT) myelography technique with coronal and oblique coronal view to demonstrate the status of the cervical nerve rootlets involved in brachial plexus injury. They discuss the value of this technique for diagnosis of nerve root avulsion compared with CT myelography with axial view.

**Methods:**

CT myelography was performed with penetration of the cervical subarachnoid space by the contrast medium. Then the coronal and oblique coronal reconstructions were created. The results of CT myelography were evaluated and classified with presence of pseudomeningocele, intradural ventral nerve rootlets, and intradural dorsal nerve rootlets. The diagnosis was by extraspinal surgical exploration with or without spinal evoked potential measurements and choline acetyl transferase activity measurement in 25 patients and recovery by a natural course in 3 patients. Its diagnostic accuracy was compared with that of CT myelography with axial view, correlated with surgical findings or a natural course in 57 cervical roots in 28 patients.

**Results:**

Coronal and oblique coronal views were superior to axial views in visualization of the rootlets and orientation of the exact level of the root. Sensitivity and specificity for coronal and oblique coronal views of unrecognition of intradural ventral and dorsal nerve root shadow without pseudomeningocele in determining pre-ganglionic injury were 100% and 96%, respectively. There was no statistically significant difference between coronal and oblique coronal views and axial views.

**Conclusion:**

The information by the coronal and oblique coronal slice CT myelography enabled the authors to assess the rootlets of the brachial plexus and provided valuable data for helping to decide whether to proceed with exploration, nerve repair, primary reconstruction.

## Background

Diagnostic imaging of brachial plexus injuries is important to locate the level of the injury, as prognosis and treatment planning depend on differentiating nerve root avulsion from lesions distal to the sensory ganglion. Preoperative imaging has previously been performed using conventional myelography, computerized tomography (CT) myelography, and magnetic resonance imaging (MRI). Sufficient contrast between the subarachnoid space and neural structure is not achieved with conventional MRI. It includes artifacts due to cerebrospinal fluid pulsation and movement by the patient [1,2]. Doi et al. [3]reported the overlapping coronal-oblique slices MRI technique, which provide clear image of the rootlets and ganglia. Accuracy of this technique is same as that of myelography/CT myelography. This technique, however, require special skill to obtain good-quality images and evaluate the images. Despite the advent of MRI, which has replaced other imaging techniques for evaluation of almost all disease of the spine, conventional myelography and CT myelography are still considered the first-choice examinations in the evaluation of brachial plexus injury [4].

Reconstructions of CT images have been applied for several assessment of disease. However, the axial CT images still remain the standard reference of the pre-operative situations of the cervical nerve roots involved in brachial plexus injury. We describe a new CT myelography technique with coronal and oblique coronal view, focusing on the shadows of the rootlets. And we discuss the diagnostic value of this technique for diagnosis of nerve root avulsion compared with traditional CT myelography with axial view.

## Methods

### Patients

Between March 2004 and December 2006, 28 patients with traumatic brachial plexus injury were examined at our institution. The group comprised 24 men and 4 women, ranging in age 15 to 56 years (mean, 29 years). 21 patients had a complete brachial plexus palsy, one had subtotal brachial plexus palsy, and four had upper brachial plexus palsy.

**Figure 1:**
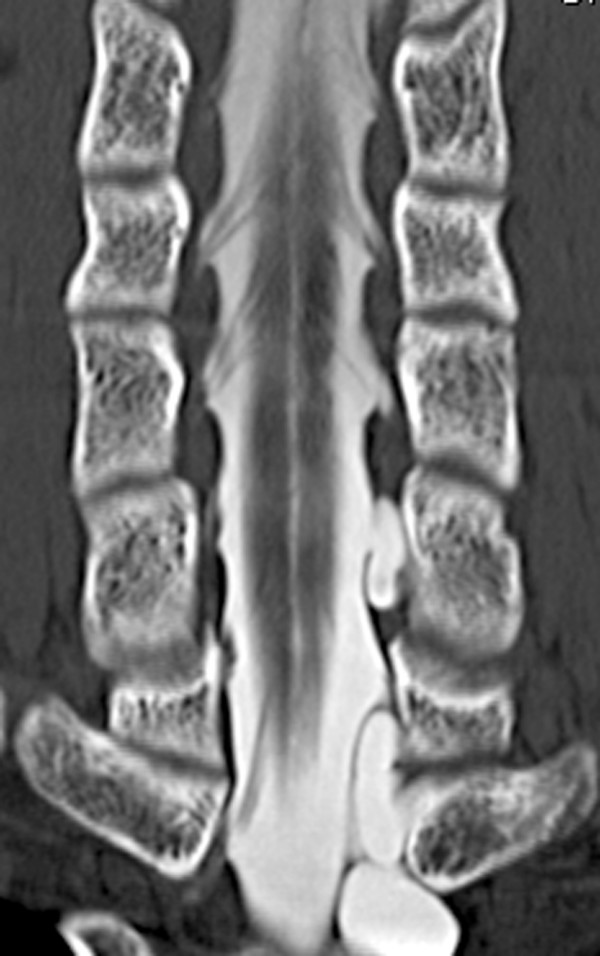
Coronal view of computerized tomography myelography visualizing the ventral rootlets. The number or size of rootlets and the connection with the cord are well visualized.

**Figure 2:**
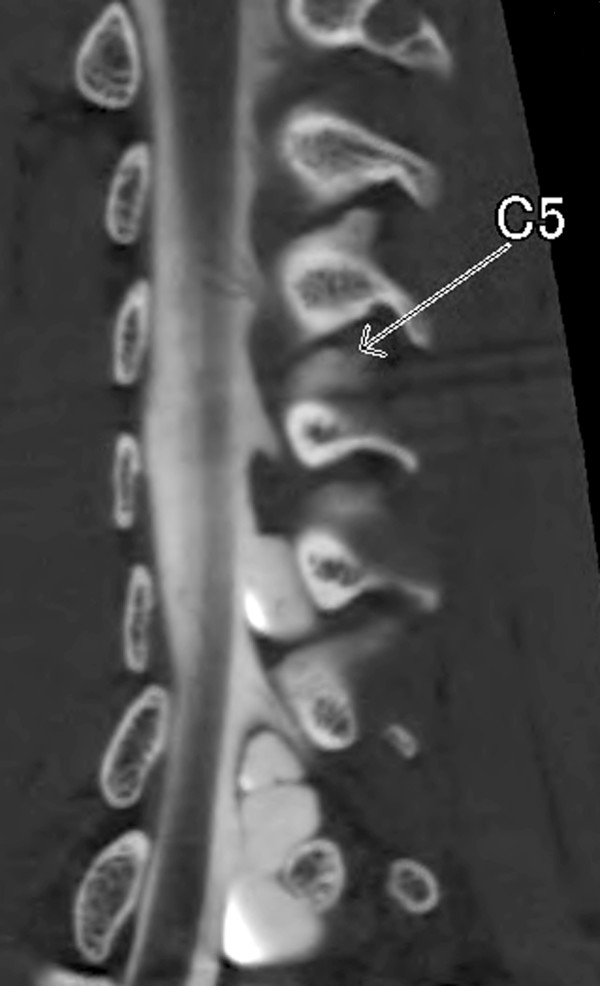
Oblique coronal view of computerized tomography myelography visualizing the dorsal rootlets.

Myelography was performed by cervical puncture employing 10 ml of water-soluble contrast medium using a concentration of 240 mg/ml Iotrolan (Isovist^(R) ^Inj. 240., Bayer Yakuhin, Ltd., Osaka, Japan). We prefer lateral C1-2 interval puncture because of our experience that details of root were better visualized than lumber puncture. Myelography was successful in all but two patients, for whom slight subdural injection degraded the quality of the CT myelography. CT myelography was performed within 10 minutes following myelography in all patients. It was performed on a 16-slice helical CT scanner (Aquilion 16, Toshiba Medical Systems Co., Ltd., Tokyo, Japan) with the following scanning protocol: Scanning parameters consisted of 16 slices with 0.5-mm x-ray beam collimation, 0.75 s of rotation time, pitch factor P = 0.938, and table feed of 10 mm·s^-1 ^and a reconstruction interval of 0.5 mm. The computed tomography dose index was 54.1 mGy. The patient was positioned supine with a small pillow placed beneath the head to flex the cervical spine. This position aligns lordotic curvature of the cervical spine in a straight line, which is very important to gain the good-quality CT myelography with coronal view. Helical images were transferred from the scanner to a workstation, Ziosoft M900 Quadra, version 3.10f (Ziosoft Inc., Tokyo, Japan). The transverse (axial) sequence was acquired to determine the direction of the ventral and dorsal roots. Coronal views (Fig. 1) were then reconstructed based on transverse slice. Oblique coronal views (Fig. 2) were by cutting parallel to the neural foramen. The best views for evaluating the dorsal root sleeves and nerve roots were the 20° to 30° anterior oblique projection. Reconstructions were successfully generated for all the patients.

In good quality CT myelogram on axial view, the ventral root and the dorsal root were clearly demonstrated in a single image. The presence of the roots was aided by comparison with the contralateral intact root. When the root of the intact side could not be identified, the affected root was not diagnosed. In some instances, the roots and the menigocele were not visualized because of epidural puncture. These images were excluded from the study.

CT myelographic diagnosis of root avulsion was based on the either both ventral and dorsal roots and the presence of a menigocele as follows: A(+); ventral root can be recognized, A(-); ventral root cannot be recognized, P(+); dorsal can be recognized, P(-);dorsal root cannot be recognized, M(+); menigocele can be recognized, M(-); menigocele cannot be recognized. When the image identify the healthy both ventral and dorsal roots without a menigocele, the findings was classified A(+)P(+)M(-). A nerve root was considered avulsed from the spinal cord when either ventral or dorsal roots were unrecognizable on axial view. On coronal and oblique coronal view, nerve roots were considered avulsed when the number or size of rootlets was decreased or the roots was absent. Image criteria for the diagnosis was based on the presence of the ventral and dorsal roots but was not the absence of either or both roots. If the findings was classified A(+)P(+)M(-), the roots were diagnosed as repairable.

The images were reviewed independently and blindly by two observers without knowledge of clinical or surgical finding. Discrepancies between the two observers were resolved by consensus. The inter-observer reliability was assessed.

The image findings were compared with the diagnosis for 57 cervical roots in 28 patients. Diagnosis was based on intraoperative findings in 25 patients and clinical findings of recovery without surgery in 3 patients. Intraoperative findings include with direct observation of the nerve roots, evoked spinal cord potentials from each nerve root, and choline acetyltransferase activity measurement [5]. The sensitivity, specificity, and diagnostic accuracy in the evaluation of the root avulsion were calculated for the 57 cervical roots in the 28 patients.

We used the Yates' chi-square test to compare the sensitivity, specificity, and diagnostic accuracy between the axial CT images and the coronal and oblique coronal CT images. The Cohen Kappa analysis was used for inter-observer reliability. The level of significance was established at p < 0.05.

## Results

Good-quality CT myelographic examinations were obtained in 49 (86%) of the 57 roots on axial view. Image quality was degraded by epidural puncture in the other 5 roots and by unrecognition of the contralateral intact root in the other 3 roots on axial view. On coronal and oblique coronal view, they were obtained in 54 (95%) roots, and image quality was degraded by epidural puncture in the other 3 roots (no statistically significant difference). These nerve roots with poor-quality image were excluded from the analysis. The kappa value for the inter-observer reliability of the axial view and the coronal and oblique coronal view was 0.91 and 0.89, respectively.

The findings with axial view were classified as repairable in 24 roots and non-repairable in 24. They showed 96% sensitivity, 83% specificity, and 90% diagnostic accuracy, with 23 true-positive findings, 20 true-negative findings, one false-positive findings, and four false-negative findings for diagnosing root avulsion.

The findings with coronal and oblique coronal view were classified as repairable in 28 roots and non-repairable in 26. They showed 100% sensitivity, 96% specificity, and 98% diagnostic accuracy, with 26 true-positive findings, 27 true-negative findings, none false-positive findings, and one false-negative findings for diagnosing root avulsion.

There was no statistically significant difference in sensitivity, specificity, and diagnostic accuracy between the two imaging technique.

## Discussion

MRI has many advantages without considerable exposure to radiation, a possible adverse reaction to contrast material, and the risk of lumber puncture. The most common findings with nerve root avulsion are traumatic meningoceles. MRI is superior to conventional myelography and CT myelography in visualizing small meningoceles, which do not fill with contrast medium in a presence of a dural scar [6]. Nerve root avulsions with no dural abnormalities and traumatic meningoceles without nerve root avulsion, however, have been reported [7]. Avulsion injury may be necessary to be evaluated on nerve rootlets.

Conventional myelography provide good anatomical depiction of root sleeves and nerve roots. But the shadows of the root are sometimes misjudged, if the concentration of the contrast medium is low. It is reported to be unreliable at the level of the fifth and sixth cervical nerve roots [7]. CT myelography is superior to conventional myelography in visualizing the nerve rootlets. It is, however, sometimes difficult to determine the exact level of the root with axial imaging, because the roots run obliquely [1]. It is difficult to detect the entire extent of root injuries with single axial slice of the images.

**Figure 3:**
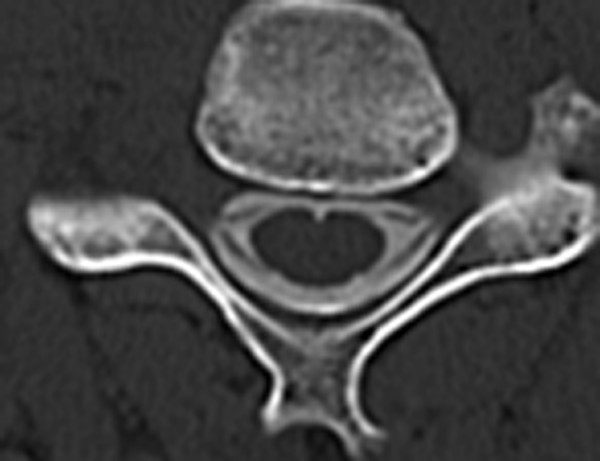
Axial view of computerized tomography myelography visualizing only a part of the rootlets.

**Figure 4:**
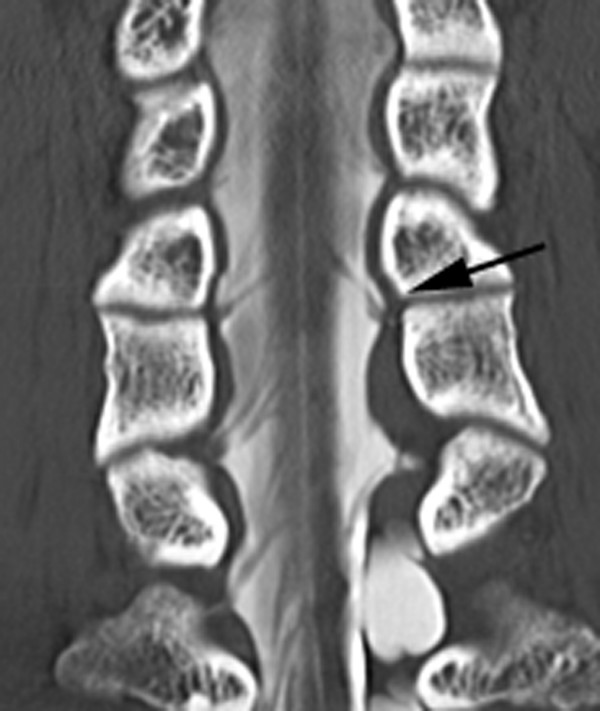
In the coronal view, decreased number or redundant of the C5 rootlets (black arrow) are well recognized.

CT myelography with axial view allows demonstration of the rootlets and also differentiation between the ventral and dorsal rootlets (Fig. 3). A particular difficulty for diagnosis with axial view, although, is assessment of the rootlets. As the spinal nerve rootlets run in oblique direction, the continuity of some nerve rootlets from the cord to the exit foramen can not be identified in axial view. Coronal and oblique coronal view was superior to conventional axial view in visualization of the number or size of rootlets and the connection with the cord, and in orientation of the exact level of the root. Coronal view visualized the whole image of the ventral rootlets, and oblique coronal view visualized the dorsal rootlets. In the case with decreased number of the rootlets or redundant rootlets (Fig. 4), intraoperative diagnosis was pre-ganglionic injury with considerable frequency. The major advantage that CT myelography with coronal and oblique coronal view adds to a good quality myelogram is the ability to identified partial injury of ventral and dorsal rootlets. We believe this technique to be useful for determining the status of the nerve rootlets and detecting nerve root avulsion, although diagnostic utility was not significant different.

In this study, we reviewed twenty-three of C5 root, seventeen of C6 root, seven of C7 root, five of C8 root, and one of Th1 root. Exploration of the all roots was not routinely performed, since the nerve graft is not effective in the lower roots. Brachial plexus exploration cannot reveal intraforaminal rootlet lesions unless laminectomy is performed. Intraoperative nerve action potentials obtained at the proximal cervical root attempt to evaluate the intraspinal status of the roots extraspinally. However, nerve action potential studies asses only the dorsal rootlets. Therefore, even a positive nerve action potential does not exclude the intradural avulsion of the ventral rootlet, because the ventral rootlets are more vulnerable than the dorsal rootlets. Choline acetyltransferase activity measurement has been applied clinically to distinguish the availability of the proximal nerve stump as a donor motor nerve during brachial plexus surgery [5]. We use choline acetyltransferase activity measurement for intraoperative diagnosis of the root avulsion in the case with discrepancies between the nerve action potential studies and the clinical or imaging diagnosis.

## Conclusion

The development of reconstructed CT myelography with coronal and oblique coronal view has provided important advantages over axial view with regard to the rootlets shadows, although diagnostic utility was not significant different. CT myelography, in spite of its invasiveness, is still indispensable for preoperative evaluation of cervical nerve root avulsion of brachial plexus injury because of its precise delineation of nerve rootlets shadows.

## Competing interests

The author(s) declare that they have no competing interests.

## Authors' contributions

HY designed the study, reviewed the images, performed myelography, helped perform surgeries, and drafted the manuscript. KD conceived the study and performed surgeries. YH reviewed the images, performed myelography, and helped perform surgeries. SS helped perform surgeries. All authors read and approved the final manuscript.
